# Supplementary material for: Increased level of FAM19A5 is associated with cerebral small vessel disease and leads to a better outcome
Source: PeerJ. 2022 Mar 8;10:e13101. doi: 10.7717/peerj.13101 (PMC8916029; doi:10.7717/peerj.13101)
Supplement: Supplemental Information 3 [file peerj-10-13101-s003.docx]

| **categorical data number code** | | |
| --- | --- | --- |
| variable | Code | meaning |
| Sex | 1 | Female |
|  | 2 | Male |
| hypertension | 0 | absence |
|  | 1 | presence |
| diabete | 0 | absence |
|  | 1 | presence |
| smoking | 0 | absence |
|  | 1 | presence |
| drinking | 0 | absence |
|  | 1 | presence |
