# Supplementary material for: Increased level of FAM19A5 is associated with cerebral small vessel disease and leads to a better outcome
Source: PeerJ. 2022 Mar 8;10:e13101. doi: 10.7717/peerj.13101 (PMC8916029; doi:10.7717/peerj.13101)
Supplement: Supplemental Information 4 [file peerj-10-13101-s004.pdf]

人序列相似家族 19 成员 A5（FAM19A5）酶联免疫吸附测定试剂盒

本试剂盒仅供体外研究使用，不用于临床诊断

使用说明书

货号：JL29722

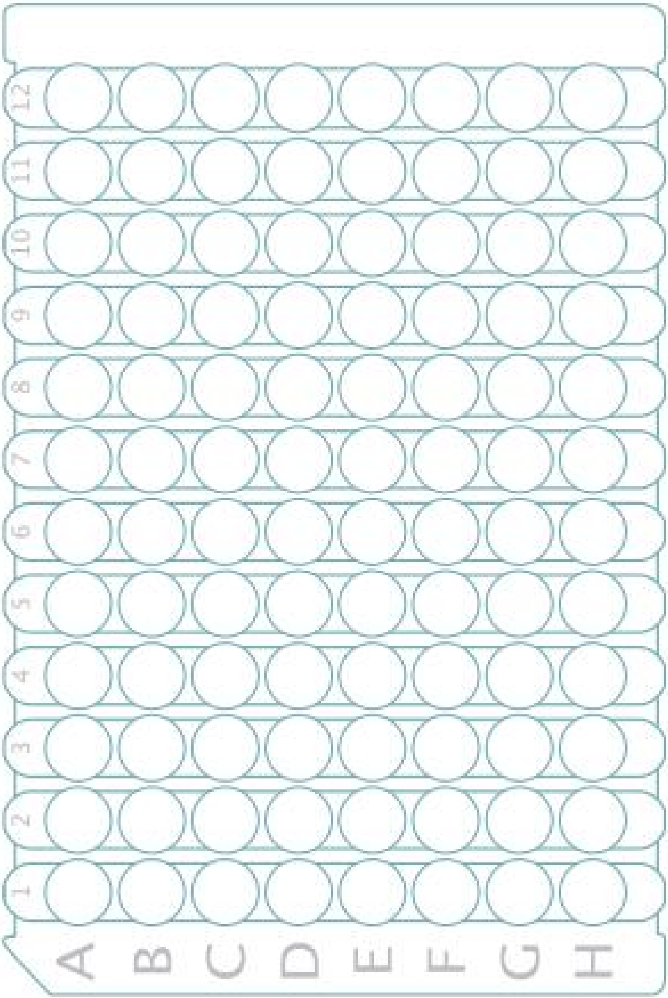
12
11
10
9
8
7
6
5
4
3
2
1
A B C D E F G H

- **本试剂盒用于体外定量检测血清、血浆、组织匀浆及相关液体样本中人序列相似家族 19 成员 A5（FAM19A5）的含量。**
- **有效期：6 个月**
- **保存条件：2-8℃**

试剂盒组成：

**名称**	**96 孔配置**	**48 孔配置**	**备注**
微孔酶标板	12 孔×8 条	12 孔×4 条	无
标准品	0.3mL*6 管	0.3mL*6 管	无
样本稀释液	6mL	3mL	无
检测抗体-HRP	10mL	5mL	无
20×洗涤缓冲液	25mL	15mL	按说明书进行稀释
底物 A	6mL	3mL	无
底物 B	6mL	3mL	无
终止液	6mL	3mL	无
封板膜	2 张	2 张	无
说明书	1 份	1 份	无
自封袋	1 个	1 个	无

备注：

1. （96T/48T）打开包装后请及时检查所有物品是否齐全。
2. 标准品浓度依次为 :6400、3200、1600、800、400、200 pg/mL
3. 经过大量正常标本检验，标本的正常浓度值均在试剂盒提供的检测范围内，实验过程中直接取 50μL 样本上样即可。当有部分样本值超过最大标准品浓度时，可用样本稀释液将标本进行适当稀释后再进行实验。

检测前准备工作：

1. 请提前 60 分钟从冰箱中取出试剂盒、样本，平衡至室温。
2. 从冰箱中取出的浓缩洗涤液可能有结晶，属于正常现象；放置室温，轻摇均匀，待结晶完全溶解后再配置洗涤液。可将 20ml 浓缩洗涤液用蒸馏水或去离子水稀释配置成 400ml 工作浓度的洗涤液，未用完的放回 4℃
3. 20×洗涤缓冲液的稀释：蒸馏水按 1：20 稀释，即 1 份 20×洗涤缓冲液加 19 份蒸馏水。

实验原理：

试剂盒采用双抗体一步夹心法酶联免疫吸附试验（ELISA）。往预先包被人序列相似家族 19 成员 A5（FAM19A5）捕获抗体的包被微孔中，依次加入标本、标准品、HRP 标记的检测抗体，经过温育并彻底洗涤。用底物 TMB 显色，TMB 在过氧化物酶的催化下转化成蓝色，并在酸的作用下转化成最终的黄色。颜色的深浅和样品中的人序列相似家族 19 成员 A5（FAM19A5）呈正相关。用酶标仪在 450nm 波长下测定吸光度（OD 值），计算样品浓度。

实验所需自备试验器材：

1. 酶标仪（450nm）
2. 高精度加样器及枪头：0.5-10uL、2-20uL、20-200uL、200-1000uL
3. 37℃恒温箱
4. 蒸馏水或去离子水

样本处理及要求：

1. **血清：**将收集于血清分离管的全血标本在室温放置 2 小时或 4℃过夜，然后 1000×g 离心 20 分钟，取上清即可，或将上清置于-20℃或-80℃保存，但应避免反复冻融。
2. **血浆：**用 EDTA 或肝素作为抗凝剂采集标本，并将标本在采集后的 30 分钟内于 2-8℃ 1000×g 离心 15 分钟，取上清即可检测，或将上清置于-20℃或-80℃保存，但应避免反复冻融。
3. **组织匀浆：**用预冷的 PBS (0.01M, pH=7.4)冲洗组织，去除残留血液（匀浆中裂解的红细胞会影响测量结果），称重后将组织剪碎。将剪碎的组织与对应体积的 PBS（一般按 1:9 的重量体积比，比如 1g 的组织样品对应 9mL 的 PBS，具体体积可根据实验需要适当调整，并做好记录。推荐在 PBS 中加入蛋白酶抑制剂）加入玻璃匀浆器中，于冰上充分研磨。为了进一步裂解组织细胞，可以对匀浆液进行超声破碎，或反复冻融。最后将匀浆液于 5000×g 离心 5~10 分钟，取上清检测。
4. **细胞培养物上清：**请 1000×g 离心 20 分钟，取上清即可检

测，或将上清置于-20℃或-80℃保存，但应避免反复冻融。

5. **其它生物标本：**1000×g 离心 20 分钟，取上清即可检测。
6. **样品外观：**样品应清澈透明，悬浮物应离心去除。
7. **样品保存：**样品收集后若在 1 周内进行检测的可保存于 4℃，若不能及时检测，请按一次使用量分装，冻存于-20℃（1 个月内检测），或-80℃（6 个月内检测），避免反复冻融，标本溶血会影响最后检测结果，因此溶血标本不宜进行此项检测。

注意事项：

1. 严格按照规定的时间和温度进行温育以保证准确结果。所有试剂都必须在使用前达到室温 20-25℃。使用后立即冷藏保存试剂。
2. 洗板不正确可以导致不准确的结果。在加入底物前确保尽量吸干孔内液体。温育过程中不要让微孔干燥掉。
3. 消除板底残留的液体和手指印，否则影响 OD 值。
4. 底物显色液应呈无色或很浅的颜色，已经变蓝的底物液不能使用。
5. 避免试剂和标本的交叉污染以免造成错误结果。

6. 在储存和温育时避免强光直接照射。
7. 平衡至室温后再打开密封袋以防水滴凝聚在冷板条上。
8. 任何反应试剂不能接触漂白溶剂或漂白溶剂所散发的强烈气体。任何漂白成分都会破坏试剂盒中反应试剂的生物活性。
9. 不能使用过期产品，不同批号组分不得混用。
10. 如果可能传播疾病，所有的样品都应管理好，按照规定的程序处理样品和检测装置。

操作步骤：

1．从室温平衡 60min 后的铝箔袋中取出所需板条，剩余板条用自封袋密封放回 4℃。

2．设置标准品孔,空白孔和样本孔，标准品孔各加不同浓度的标准品 50μL。

3．样本孔中加待测样本 50μL；空白孔加样本稀释液 50μL。

4．空白孔，标准品孔和样本孔中每孔加入辣根过氧化物酶（HRP）标记的检测抗体 100μL，用封板膜封住反应孔，37℃水浴锅或恒温箱温育 60min。

5．弃去液体，吸水纸上拍干，每孔加满洗涤液（350μL），静置 1min，甩去洗涤液，吸水纸上拍干，如此重复洗板 5 次（也可用洗板机洗板）。

6．每孔加入底物 A、B 各 50μL，37℃避光孵育 15min。

7．每孔加入终止液 50μL，15min 内，在 450nm 波长处测定各孔的 OD 值。

实验结果计算：

绘制标准曲线：在 Excel 工作表中，以标准品浓度作横坐标，对应 OD 值作纵坐标，绘制出标准品线性回归曲线，按曲线方程计算各样本浓度值。

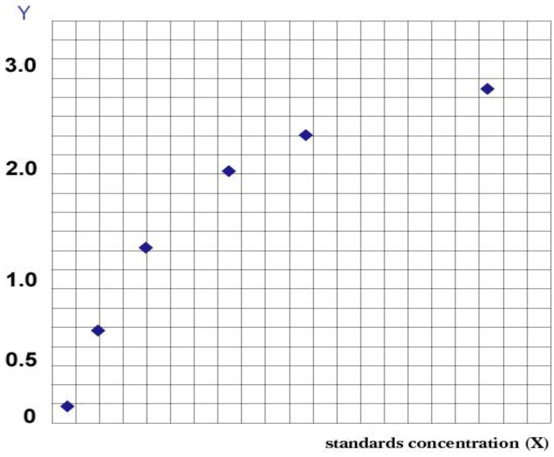

（此图仅供参考）

试剂盒性能：

1. 检测范围：200 pg/mL – 6400 pg/mL。
2. 灵敏度：最低检测浓度小于 10 pg/mL。
3. 特异性：不与其它可溶性结构类似物交叉反应。
4. 重复性 :板内变异系数小于 9% ,板间变异系数小于 11% 。

技术小提示：

1. 当混合或重溶蛋白溶液时，尽量避免起沫。
2. 为了避免交叉感染，配置不同浓度标准品、上样、加不同试剂都需要更换枪头。另外不同试剂请分别使用不同的移液槽。
3. 每次孵育时，请正确使用封板胶可保证结果的准确性。
4. 混合后的显色底物在上板前应为无色，请避光保存；加入微孔板后，将由无色变成不同深度的蓝色。
5. 终止液上板顺序应同显色底物上板顺序一致；加入终止液后，孔内颜色由蓝变黄；若孔内有绿色，则表明孔内液体未混匀；请充分混合。

说明：

1. 由于现有条件及科学技术水平尚不能对所有供货商提供的所有原料进行全面的鉴定与分析，本产品可能存在一定的质量技术风险。
2. 最终的实验结果与试剂的有效性、实验者的相关操作以及当时的实验环境密切相关，请务必准备充足的标本备份。
3. 不同批次的同一产品可能会有少许差别，如：检测限、灵敏度以及显色时间等，请依据试剂盒内说明书进行实验操作，网站电子版说明书仅作参考。
4. 使用化学裂解液制备的组织匀浆或细胞提取液可能会由于某些化学物质的引入导致 ELISA 实验结果偏差。
5. 若样本为细胞培养上清，因该类样本干扰因素较多，如：细胞状态、细胞数量、采样时间等，所以可能存在检测不出的情况。
6. 某些天然蛋白或重组蛋白，包括原核及真核重组蛋白，可能因为与本产品所使用的检测抗体及捕获抗体不匹配，而不被检测出。

咨询电话： 400-0066-400

传　　真： 021-55660885

电子邮箱： shjlsw@163.com

网　　址： www.jonln.com
